# Phylogenomic and Microsynteny Analysis Provides Evidence of Genome Arrangements of High-Affinity Nitrate Transporter Gene Families of Plants

**DOI:** 10.3390/ijms222313036

**Published:** 2021-12-03

**Authors:** Normig M. Zoghbi-Rodríguez, Samuel David Gamboa-Tuz, Alejandro Pereira-Santana, Luis C. Rodríguez-Zapata, Lorenzo Felipe Sánchez-Teyer, Ileana Echevarría-Machado

**Affiliations:** 1 Unidad de Bioquímica y Biología Molecular de Plantas, Centro de Investigación Científica de Yucatán A.C., Mérida 97205, Mexico; normigzr@gmail.com; 2 Unidad de Biotecnología, Centro de Investigación Científica de Yucatán A.C., Mérida 97205, Mexico; sd.gamboa.t@gmail.com (S.D.G.-T.); lcrz@cicy.mx (L.C.R.-Z.); 3 Conacyt-Unidad de Biotecnología Industrial, Centro de Investigación y Asistencia en Tecnología y Diseño del Estado de Jalisco, Guadalajara 44270, Mexico; apereira@ciatej.mx

**Keywords:** comparative genomics, gene evolution, high-affinity nitrate transporters, land plants, nitrate absorption

## Abstract

Nitrate transporter 2 (NRT2) and NRT3 or nitrate-assimilation-related 2 (NAR2) proteins families form a two-component, high-affinity nitrate transport system, which is essential for the acquisition of nitrate from soils with low N availability. An extensive phylogenomic analysis across land plants for these families has not been performed. In this study, we performed a microsynteny and orthology analysis on the NRT2 and NRT3 genes families across 132 plants (*Sensu lato*) to decipher their evolutionary history. We identified significant differences in the number of sequences per taxonomic group and different genomic contexts within the NRT2 family that might have contributed to N acquisition by the plants. We hypothesized that the greater losses of NRT2 sequences correlate with specialized ecological adaptations, such as aquatic, epiphytic, and carnivory lifestyles. We also detected expansion on the NRT2 family in specific lineages that could be a source of key innovations for colonizing contrasting niches in N availability. Microsyntenic analysis on NRT3 family showed a deep conservation on land plants, suggesting a high evolutionary constraint to preserve their function. Our study provides novel information that could be used as guide for functional characterization of these gene families across plant lineages.

## 1. Introduction

Most of the nitrogen (N) present in the soil (>98%) is organic matter, which is mostly not available directly to plants, and its conversion to assimilable inorganic N forms occurs through biological processes involving soil microorganisms. Nitrate (NO_3_^−^) is the main form of N in agricultural soils, where its availability can become a major limitation in agricultural productivity [1,2,3]. The nitrate concentration in soils is highly variable, both spatially and temporally, ranging from micromolar amounts to 70 mM depending on the type of soil, climate, ecological characteristics, microbial activity, land use, and the addition of fertilizers, among other factors [4]. Additionally, NO_3_^−^ acts as a signal molecule that regulates seed germination, induces leaf expansion, regulates lateral root development, and coordinates the expression of genes related to nitrate assimilation as well as other nutrients [1,5]. Plants have developed transport systems with different affinities to absorb NO_3_^−^ from the roots, distribute it, and store it. Among these transport systems are those with low affinity (LATS), which operate when external concentrations of NO_3_^−^ are greater than 1 mM, and those with high affinity (HATS), which operate at micromolar concentrations of NO_3_^−^ [6]. HATS may be key to nitrate absorption under conditions of low availability, where HATS activity may have hundreds of times more affinity for N, thereby supplanting LATS activity [7,8].

HATS is carried out by transporters of the NRT2 (nitrate transporter 2) family (nitrate/nitrite porter, NNP) [9,10]. To date, it is known that HATS comprise less numerous families than LATS [11,12], although most of their members have yet to be functionally characterized. Some proteins are exceptions to this rule and may have HATS and LATS properties, such as OsNRT2.4 from *Oryza sativa* [13], and others, such as CrNRT2.3 from *Chlamydomonas reinhardtii*, which has LATS activity rather than HATS activity [14]. In plants, the members of NRT2 are identified through the characteristic domain of the NRT2/NNP proteins [15] as a subfamily of the MFS (major facilitator superfamily). The MFS recently expanded to the MFS superfamily (MFSS), which groups together secondary transporters present in all living beings, and their common ancestor in plants has been reported in green algae [16,17]. It is known that HATS is a two-component system, in which the NRT2 proteins, except AtNRT2.7 from *Arabidopsis thaliana* [18] and OsNRT2.3b from *O. sativa* [19], require association with a third family of proteins, called NRT3 (formerly NAR2, proteins associated with nitrate assimilation) to form active HATS [20,21,22].

To date, seven NRT2 members (AtNRT2.1–AtNRT2.7) have been reported in *A. thaliana* [23]. Four of these NRT2 members (AtNRT2.1, 2.2, 2.4 and 2.5) are components of nitrate uptake from the root and are located on the plasma membrane, with AtNRT2.1 being the main transporter induced for the uptake of N in the root. AtNRT2.1 is located in epidermal and cortical cells, where the highest nitrate uptake occurs [23,24]. In contrast, AtNRT2.2 is only expressed in a compensatory manner when the function of NRT2.1 is lost [11,23]. AtNRT2.4 and AtNRT2.5 are expressed in the absence of N although AtNRT2.4 also participates in the loading of NO_3_^−^ towards the phloem and AtNRT2.5 in the absorption and remobilization process in adult plants subjected to severe N deficiency [11,25,26]. Unlike these four transporters, AtNRT2.7 is found in the tonoplast, and this subcellular localization may explain its lack of interaction with NAR2. AtNRT2.7 carries out the supply of nitrate to the seeds and its storage in the vacuoles [18]. In contrast, the transporters AtNRT2.3 and AtNRT2.6 have yet to be characterized as nitrate transporters in plants [9,11]. However, it has been determined that AtNRT2.3 can modulate nitrate transport in *Xenopus* oocytes [23] and that AtNRT2.6 participates together with AtNRT2.5 in *Rhizobacterium*-induced lateral root growth [27]. In addition to their function as nitrate transporters, these proteins participate in signaling mechanisms to regulate the development of lateral roots [2,11] and in bacterial sensing [27].

One of the biggest challenges facing quantitative genetics and molecular evolution is deciphering the way different genomes interact to originate a unique phenotype through mechanisms such as horizontal gene transfer and polyploidy [28]. In this study, the gene expression of NRT2 and NRT3 can be considered as epigenetics as a function of the availability of N, which also obeys the forces of transgenerational epigenetic inheritance understood as complex traits of tolerance to inherited abiotic stress and can be studied through heritability estimates to evaluate the effects of climate change [29] or genotype-pathogen–environment interactions [30].

Regarding the NRT3 family, which comes from green algae, the association with NRT2 has been demonstrated in *A. thaliana* [20,22] and *O. sativa* [31]. These proteins are identified by the presence of a characteristic domain of NAR2 proteins [8,20,22]. In addition to Arabidopsis, six NRT2 and five NRT3 have been identified in *Populus trichocarpa* [32], three NRT2 and one NRT3 in *Ananas comosus* [33], four NRT2 in *Zea may* [34], 17 NRT2 in *Brassica napus* L. [7], and 10 NRT2 and three NRT3 in *Hordeum vulgare* L. [35]. Functional and molecular studies of high-affinity transporters have been partially carried out in other species, such as *Lotus japonicus* and *Medicago truncatula* [36], *Zostera marina* [37], *Triticum aestivum* [38], *Capsicum chinense* [21], *Spinacia oleracea* L. [17], and *Malus domestica* [39], among others.

The genetic diversity of the NRT2 and NRT3 genes can be exploited for agriculture either by marker-assisted breeding or through genetic engineering. The diversity of functions and nomenclature between species makes it difficult to select candidate genes for these purposes, which could be facilitated with studies approached from an evolutionary point of view. Von Wittgenstein et al. [40] performed phylogenetic analysis to reconstruct the evolutionary history of NRT2 through 20 genomes of terrestrial plants, complemented with two species of green algae. From this study, two clades of NRT2 in angiosperms were suggested; clade I contained members for all angiosperms analyses, while clade II only had sequences from eight of the 20 genomes studied. These authors concluded the need to include additional taxa from other taxonomic groups, such as gymnosperms, to assess the complete evolutionary history of this transporter system.

It is important to mention that variability in sequence of genes, including coding and flanking regions, as well as the chromosomal blocks organization could determine the existence of diverse haplotypes as reported in wheat for NRT2 [41]. Once the variability among genes is determined, it may be necessary to evaluate the linkage disequilibrium (LD) at population level to evaluate a significant deviation and indicative of selective pressure [42]. To our knowledge, only few reports of LD of the NRT2 and NRT3 genes of high-affinity nitrate transporters has been published; however, it can be observed that, in the mapping of these genes, each chromosome presented variability in the amount of the linkage blocks as well as in degrees of association between the genes that encode these transporters [35].

With data from multiple genomes, it is possible to estimate the positional conservation or the genomic context within these gene families with a synteny approach. Synteny (the analysis of the relative gene order) provides information about genome rearrangements between genomes and provides strong evidence to define orthology relationships. Comparative analysis coupled to microsynteny would help to understand the evolutionary trajectory of genes and ultimately of key innovations into species [43,44,45,46,47]. Synteny studies have been conducted for the analysis of nitrate transporters in a few species, comparing them in a limited way with the model species *A. thaliana* [7,33]. However, the analysis of multiple genomes would provide a more solid view of the evolutionary process [48].

To visualize patterns of an entire syntenic relationship from multigene families across multiple genomes, a microsynteny network analysis was recently proposed. This approach allows a rapid analysis and visualization of synteny relationships and obtains new knowledge from it about the evolutionary history of a target gene family [43,45,46,49]. In this study, a phylogenomic analysis was carried out on 854 proteins from the NRT2 family and 299 proteins from the NRT3 family across 132 plant (*Sensu lato*) genomes. The main goal of our study was to provide an overview of the evolution of these proteins, representing an important target in agricultural biotechnology to improve the efficiency of N usage, and ultimately to contribute to the reduction in the application of N fertilizer. Our study provides an overview of the rearrangements process of the genetic structure that contain NRT2 and NRT3 genes in different taxonomic groups, which allows establishing more precise relationships between transporters from different linages with contrasting adaptive responses.

## 2. Results

### 2.1. NRT2 and NRT3 Sequences Identification

**Figure 1:**
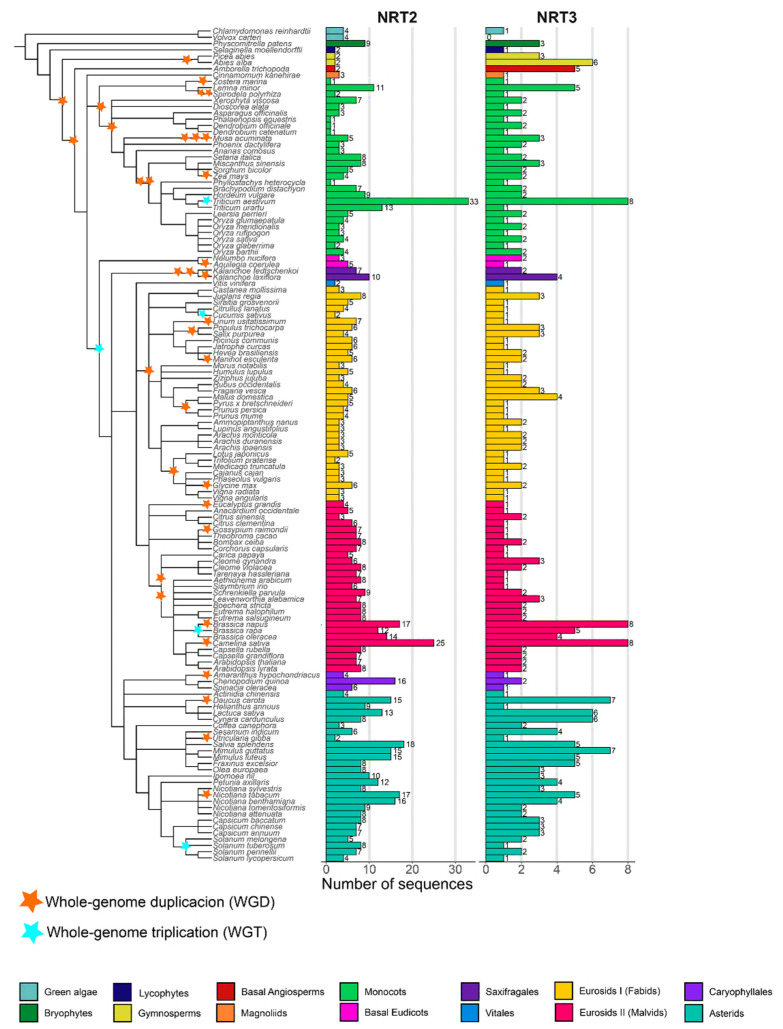
NRT2 (nitrate transporter 2) and NRT3 (nitrate-assimilation-related 2, NAR2) genes number and phylogenetic analysis of the species studied. Phylogenetic tree of the 132 plant species studied in 14 taxonomic groups: green algae (2), bryophytes (1), lycophytes (1), gymnosperms (2), basal angiosperms (1), magnoliids (1), monocots (28), basal eudicots (2), saxifragales (2), vitals (1), eurosides I (fabids; 34), eurosides II (malvids; 27), Caryophyllales (3), and asterids (27). Number of sequences retrieved considering the HMM profile with a coverage ≥60% of protein domains. The tree was visualized using iTOL (http://itol.embl.de/) (accessed on 21 Jul 2020). The duplications (WGD) and triplications (TGD) of the entire genome are represented by yellow and blue triangles, respectively (based on Angiosperm Phylogeny Website).

Nitrate transporter sequences that belong to the protein families NRT2 and NRT3 were identified in 132 species distributed in 13 taxonomic groups: green algae (2), bryophytes (1), lycophytes (1), gymnosperms (2), basal angiosperms (1), magnoliids (1), monocots (28), basal eudicots (2), saxifragales (2), vitals (1), eurosides I (34 fabids), eurosides II (27 malvids), Caryophyllales (3), and asterids (27) (Table S1), spanning an evolutionary history of approximately 450 my. The phylogenetic relationship of the plant species showed a distribution of taxa similar to the evolutionary taxonomic classification described for plants in the Angiosperm Phylogeny Website (APG IV; Figure 1).

By using the HMM profiles, it was possible to recover a total of 1153 sequences (Table S2, Figure S1), of which 854 and 299 sequences were from the NRT2 and NRT3 families, respectively. Under the criteria defined in this study, members of NRT2 were present in all the species studied, while NRT3 was not found in the alga *Volvox carteri* (Figure 1).

The average number of NRT2 sequences was 6.5 sequences per species. Only two species had more than 20 NRT2 sequences, *T. aestivum* and *Camelina sativa*, with 33 and 25 sequences, respectively. In contrast, 46% of the species presenting 5–9 sequences, 40% of the species presented <5, and 12% ranged from 10–19 sequences. Four sequences were identified in green algae, the most ancestral taxa studied, while in bryophytes, lycophytes, gymnosperms, and *A. trichopoda,* only two sequences were identified except for *Physcomitrella patens*, which presented nine sequences (Figure 1).

In the monocots, *Z. marina*, *Dendrobium catenatum*, *Dendrobium officinale*, *Phalaenopsis equestris*, and *Phyllostachys heterocycla*, a single sequence was identified, in contrast with the 33 sequences identified in the hexaploidy *T. aestivum*. For the fabids, the highest number of NRT2 sequences was found in the Juglans regia with eight sequences, while the lowest number was found in the *Cucumis sativus* and *Trifolium pratense*, with two sequences each. In the malvids, a minimum of three sequences were recovered from *Citrus sinensis*, and a maximum of 25 were recovered from *C. sativa*. In Caryophyllales, a maximum of 16 sequences was identified in *Chenopodium quinoa*, while in the asterids, the sequence number ranged from two sequences in *Utricularia gibba* up to 18 sequences in *Salvia splendens* (Figure 1).

The NRT3 family was three times less abundant in average regarding the NRT2 family except for two gymnosperms, the basal angiosperm *A. trichopoda* and the monocot *D. officinale*. The average number of NRT3 sequences per genome was two. By taxonomic group, a maximum of four sequences were identified in the fabids (*Malus domestica*) and saxifragales (*Kalanchoe laxiflora*), seven sequences in the asterids (*Mimulus guttatus* and *Daucus carota*), and eight sequences in the monocots (*T. aestivum*) and the malvids (*B. napus* and *C. sativa*). In summary, 28% of the species studied presented three or more NRT3 sequences compared to 71% that presented fewer than three sequences (Figure 1).

### 2.2. Microsynteny Analysis of Nitrate Transports

**Figure 2:**
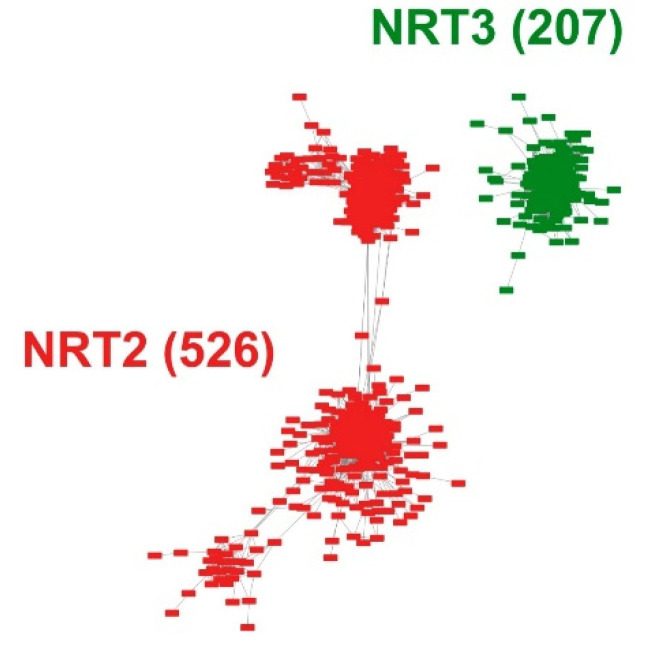
Pairwise syntenic networks of the NRT2 and NRT3 transporter sequences. The synteny relationships were obtained through the MCScanX program and visualized through the Cytoscape program. Nodes represent protein-coding genes, and edges represent pairwise synteny relationships.

Intending to develop an entire microsynteny network, all the selected sequences were pairwise compared to obtain a matrix of all the inter- and intra-genomic microsyntenic blocks (collinear regions). From this matrix, only 526 (61%) and 207 (69%) sequences met the collinearity criteria of the total recovered sequences for NRT2 and NRT3, respectively (Table S3). From this, a total of 18,016 edges (pairwise syntenic relationships) were detected. The nodes were grouped into two microsynteny networks, one for each family, without finding interactions between them (Figure 2). Likewise, of the total interactions, 12,555 and 5461 were obtained for the NRT2 and NRT3 nodes, respectively (Table S4).

The microsyntenic networks were clustered to find highly connected communities. The community was defined as a group of genes within each gene that has ≥3 pairwise syntenic connections. Under this criterion, only 502 NRT2 and 200 NRT3 sequences were grouped in syntenic communities (Table S3).

**Figure 3:**
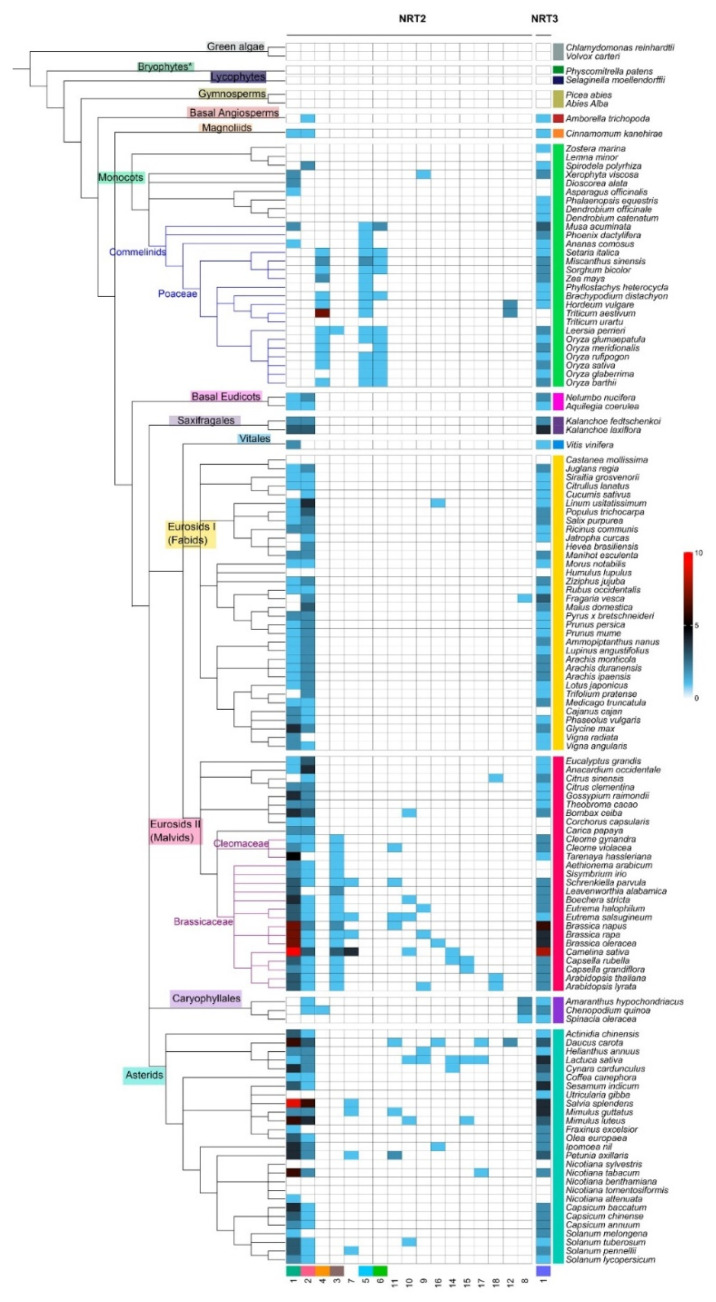
Heat map with the distribution of the syntenic communities of NRT2 genes in the species. Phylogenetic representation of the plant species and the distribution of the gene number belonging to each community (columns identified with number at the bottom) for each species (rows) in the different taxonomic groups (identified with different colors). The blue cells represent the presence of genes into syntenic communities (quantity per color scale) in the different species.

**Figure 4:**
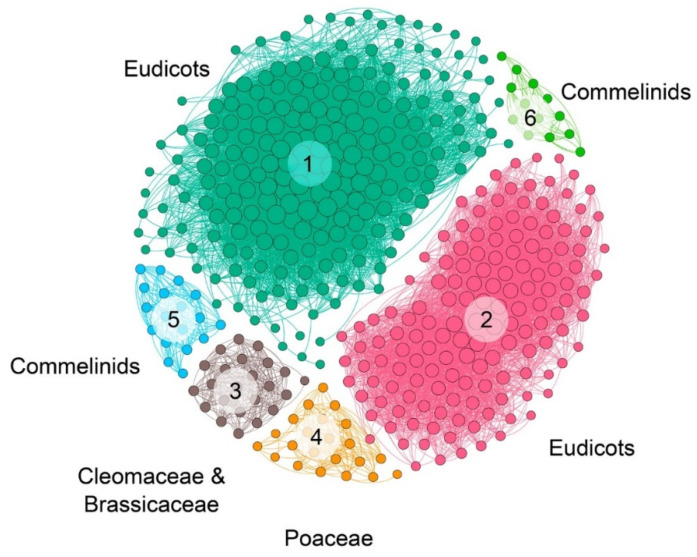
NRT2 microsyntenic gene network of the six main communities found in plant. The size of each node corresponds to the number of genes each community contains (larger nodes have more connections). The different communities are colored and labeled.

**Figure 5:**
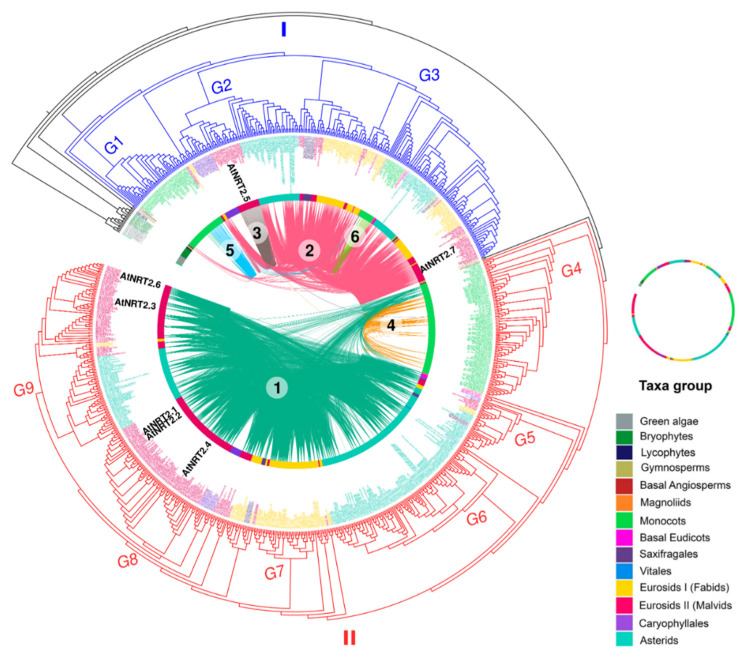
Phylogenetic tree of NRT2 gene family. From the outside to the center: tree of maximum circular likelihood of the NRT2 genes in which syntenic relationships were detected. The intermediate ring represents the different taxonomic groups where the genes are represented with different colors. Concentric lines represent the different communities of syntenic genes (distinguishable by color and assigned number) and the connections of each pair of genes are represented using curved lines. Syntenic connections are established between the same community or between different communities. The image depicts the location of NRT2 genes of *A. thaliana*.

The syntenic communities were represented in a heat map, where 17 NRT2 communities (Com1–17_NRT2) and one NRT3 community (Com1_NRT3) were identified (Figure 3 and Figure 4). For the subsequent phylogenomic analyses, the six Com_NRT2 that were most abundant (Com1–6_NRT2) were considered (Figure 4 and Figure 5).

In the comparative analysis of NRT2 and NRT3 sequences, no syntenic relationship was revealed in green algae, the bryophytes, and the gymnosperms, and collinearity was identified from the basal angiosperm.

The Com1_NRT2 (218 genes in 86 species) and Com2_NRT2 (146 genes in 85 species) communities were present mainly in eudicots (72.5%); Com3_NRT2 was specific for the Cleomaceae and Brassicaceae families and present in Poaceae *Leersia perrieri*, representing 4.6% of the sequences in synteny. The Com4_NRT2, Com5_NRT2, and Com6_NRT2 communities were mostly represented in the monocots (representing 10.5% of the sequences) (Figure 3 and Figure 4), and the 12 minority communities (from Com7_NRT2 to Com18_NRT2) represented 12.3% of the total sequences into communities.

Syntenic relationships for NRT2 were not revealed in the following taxa: monocots (*Z. marina*, *Lemna minor*, *P. equestris*, *D. officinale*, *D. catenatum*, *T. urartu*), fabids (*Castanea mollissima*, *Humulus lupulus*), Caryophyllales (*Spinacia oleraceae*), and asterids (*U. gibba*, *Nicotiana sylvestris*, *N. benthamiana, N. tomentosiformis*) (Figure 3). For NRT3 genes, no syntenic relationships were detected in five members of the monocots (*L. minor*, *Dioscorea alata*, *Asparagus officinalis*, *T. aestivum*, *T. urartu*), four of the fabids (*C. mollissima*, *Hevea brasiliensis*, *H. lupulus*, *Canus cajan*), four of the malvids (*Corchorus capsularis*, *Carica papaya*, *Aethionema arabicum*, *Sisymbrium irio*), and four of the asterids (*N. sylvestris*, *N. benthamiana*, *N. tomentosiformis*, *N. attenuata*) (Figure 3).

Regarding the abundance of syntenic sequences per species, a greater number of sequences of Com1_NRT2 were revealed in some Brassicacea species, mainly in *C. sativa* (13 sequences), and in the asterid specie, *S. splendens* (nine) (Figure 3 and Table S3).

In the phylogenetic analysis of NRT2 sequences, the genes of green algae and one of the genes of the aquatic plant *L. minor* were located in the most external position, from which bryophytes and vascular plants diverged, to later separate the sequences of the Spermatophyta. From the flowering plants, the NRT2 genes could be clearly divided into two major clades (clade I and clade II) (Figure 5).

Clade I grouped 313 NRT2 sequences that included members of all the genomes of flowering plants studied except *Vitis vinifera*. By taxonomic group, the sequences of this clade were composed of two gymnosperms, one basal angiosperm, two magnoliids, 59 monocots, four basal eudicots, ten saxifragales, 70 fabids, 62 malvids, 15 Caryophyllales, and 68 asterids. Approximately 77% of these sequences were grouped into four syntenic communities: Com2_NRT2, Com3_NRT2, Com5_NRT2, and Com6_NRT2 (Figure 5).

Clade I could be subdivided into three large subclades (G1–G3). Interestingly, the G1 subclade, grouping 43 sequences, was exclusively made up of monocot sequences, 18 of which were grouped into Com5_NRT2 although one member belonged to Com2_NRT2 and two to Com6_NRT2. In this group, the characterized sequences OsNRT2.3 (osa_LOC_Os01g50820osa) of *O. sativa*, HvNRT2.1 (HORVU3Hr1G066090) of *H. vulgare*, AcNRT2.3 (aco_016541) of *A. comosus*, and ZmNRT2.5 (zma_GRMZM2G455124) of *Z. mays* were identified (Figure 5).

The G2 subgroup exclusively grouped members of the evolutionarily more recent eudicots, the malvids (22), Caryophyllales (15), and asterids (42). Only three genes of Caryophyllales belonged to syntenic communities, two to Com2_NRT2, and one to Com4_NRT2. All the malvid genes belonged to Com3_NRT2, while 23 of asterid were from Com2_NRT2. AtNRT2.5 (ath_AT1G12940) of *A. thaliana* was identified in this subgroup (Figure 5).

Subgroup G3 grouped sequences from different evolutionary lineages, including magnoliids (2), monocots (15), and eudicots (144). Within the eudicot sequences, four basal eudicots, 10 saxifragales, 66 fabids, 38 malvids, two Caryophyllales, and 42 asterids were located. Genes within this group, except monocots, belonged exclusively to Com2_NRT2. Most of the monocot sequences belonged to Com6_NRT2 although one member of this lineage belonged to Com5_NRT2 and another to Com2_NRT2 itself. In this subgroup, the characterized sequences AtNRT2.7 (ath_AT5G14570) from *A. thaliana*; AcNRT2.2 (aco_016540) of *A. comosus* MtNRT2.3 (mtr_8g069775) from *M. truncatula*; HvNRT2.10 (HORVU6Hr1G098550) of *H. vulgare*; and PtNRT2.5a (ptr_015g081300), PtNRT2.5b (ptr_015g081500), PtNRT2.5c (ptr_012g087700), and PtNRT2.7 (ptr_001g348300) of *P. trichocarpa* were identified (Figure 5).

The two gymnosperm genes as well as the basal angiosperm gene that were identified in this clade were located outside of and sisters to all the subgroups. Of these, only the basal angiosperm gene clustered in Com2_NRT2. Three and two members of fabids and malvids, respectively, were located outside of and were sisters to G2 and G3, and all of them belonged to Com2_NRT2 (Figure 5).

Clade II contained more sequences than clade I, including 541 NRT2 sequences from which two belonged to gymnosperms, one basal angiosperm, one magnoliids, 93 monocots, four basal eudicots, seven saxifragales, two vitales, 77 fabids, 162 malvids, 11 Caryophyllales, and 181 asterids. Sequences of nine flowering plants were not identified within this clade, seven of which belonged to the monocots (*Z. marina*, *L. minor*, *Spirodela polyrhiza*, *P. equestris*, *D. catenatum*, *Phyllostachys heterocycla,* and *O. glaberrima*), one to the fabids (*Trifolium pratense*), and another to the asterids (*U. gibba*) (Figure 5).

Approximately 37% of these sequences were grouped into two syntenic communities, Com4_NRT2, which contained exclusively monocot genes, and Com1_NRT2, which contained mostly eudicot genes, although eight monocot genes were also grouped in this community (Figure 5).

Clade II could be subdivided into six major subclades (G4–G9). All the monocot sequences were grouped into the G4 subclade, which was exclusive for this taxonomic group, and contained the characterized genes OsNRT2.1 (osa_LOC_Os02g02170osa) and OsNRT2.2 (osa_LOC_Os02g02190osa) of *O. sativa*; HvNRT2.3 (HORVU6Hr1G005580), HvNRT2.4 (HORVU6Hr1G005590), HvNRT2.5 (HORVU6Hr1G005600), HvNRT2.6 (HORVU6Hr1G005720), HvNRT2.7 (HORVU6Hr1G005770), HvNRT2.8 (HORVU6Hr1G005780), and HvNRT2.9 (HORVU6Hr1G005930) of *H. vulgare*; AcNRT2.1 (aco_010201) of *A. comosus*; and ZmNRT2.2 (zma_GRMZM2G010251), ZmNRT2.3 (zma_GRMZM2G163866), and ZmNRT2.1 (zma_GRMZM2G010280) of *Z. may*. Thirty-five sequences were grouped in G5, which included one from the basal eudicots, one from the vitals, four from the fabids, seven from the malvids, and 19 from the asterids. G6 was composed solely of members of the asterids (108 sequences), while G7 was exclusive for the fabids (42 sequences). G8 included 88 sequences, 11 from Caryophyllales and the rest from the malvids, and in this group, the AtNRT2.1 (ath_AT1G08090), AtNRT2.2 (ath_AT1G08100), and AtNRT2.4 (ath_AT5G60770) genes from *A. thaliana* and the BnNRT2.1a (bna_C08g02430), BnNRT2.1b (bna_Anng40490), BnNRT2.1c (bna_Anng3550), BnNRT2.1d (bna_A06g04560), BnNRT2.1e (bna_A06g04570), BnNRT2.1f (bna_A09g49050), BnNRT2.1g (bna_C08g43380), BnNRT2.2a (bna_C08g43370), BnNRT2.2b (bna_A09g49040), BnNRT2.4a (bna_Anng28170), and BnNRT2.4b (bna_A09g35990) genes from *B. napus* L. were identified. Finally, the malvid (63) and asterids (55) sequences were mostly located in G9 although three fabid sequences were also grouped here. In this last subgroup, AtNRT2.3 (ath_AT5G60780) and AtNRT2.6 (ath_AT3G45060) from *A. thaliana* and BnNRT2.3a (bna_A09g54030) and BnNRT2.3b (bna_A10g13570) from *B. napus* L. were identified.

As in clade I, the two genes from the gymnosperms as well as the basal angiosperm were located outside and sister of all the subgroups. These genes were not clustered in syntenic communities. Other smaller subgroups were identified that were located outside the larger subgroups. For example, outside of and sister to all the major subgroups, a sequence of magnoliids and three sequences of basal eudicots were identified; on the outside of and sister to the subgroups G6–G9, a sequence of vitales was located. On the outside of and sister to G7–G9, 13 sequences were identified: 12 fabids and one malvids; on this small subgroup, PtNRT2.4a (ptr_009g008500) and PtNRT2.4b (ptr_009g008600) of *P. trichocarpa* were located. Finally, on the outside of and sister to the G8 and G9 subgroups, the following sequences were grouped into three small subgroups: (1) 19 sequences: two malvids, four saxifragales, and 13 fabids; (2) seven malvids; and (3) five malvids.

Few syntenic connections were detected between members of the two different clades, and these connections only occurred between subgroups G2 and G4, as mentioned above.

**Figure 6:**
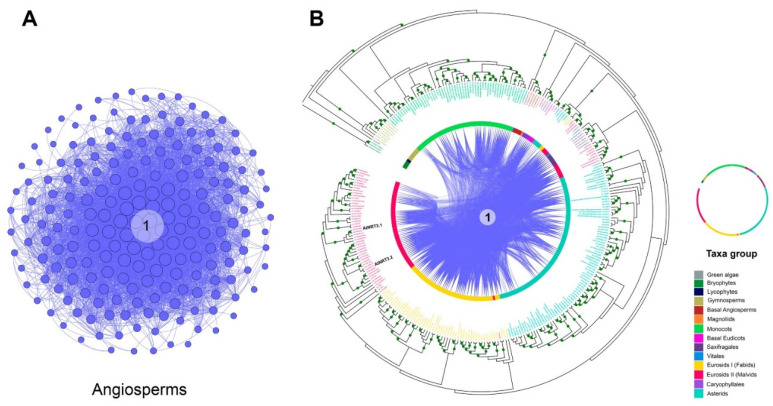
Analysis of the microsyntenic and phylogenomic network of NRT3 genes in taxonomic groups. (**A**) Network of NRT3 genes from the only community in which synteny relationships were determined. (**B**) Circular phylogenetic tree. From the outside to the center: tree of maximum circular likelihood of the NRT3 genes in which syntenic relationships were detected. The intermediate ring represents the different taxonomic groups where the genes present are represented with different colors. Concentric lines represent the connections of each gene pair using curved lines. The image depicts the location of some *A. thaliana* genes.

According to phylogenetic analysis of NRT3 sequences, a monophyletic origin was clearly observed for this protein family. Genes belonging to the bryophytes, lycophyte, gymnosperms, basal angiosperm, and monocots were grouped into separate and specific subclades for each taxon. The five basal angiosperm genes were grouped into a subclade along with the single gene of asterid *U. gibba*. All six genes of the saxifragales clustered together into a subclade with that of vitales, while those of Caryophyllales clustered together in another subclade with that of the magnoliid (Figure 6).

Fabid sequences were divided into two subclades: one that encompassed the vast majority of the sequences (53), being practically specific for this taxonomic group—with the exception of one sequence from the malvids *Eucalyptus grandis*—and the other subgroup that clustered two sequences of this taxon with four malvid and five asterid sequences. In addition to the four sequences described in the previous subgroup, 50 malvid sequences were grouped in a subclade specific to them, where AtNRT3.1 (ath_AT5G50200) and AtNRT3.2 (ath_AT4G24730) of *A. thaliana* were identified. In a third subgroup, eight malvid sequences were grouped together with all the saxifragales (five) and the vitales (one). A specific subclade for the asterids grouped 85 sequences in addition to the two subclades described above, in which sequences from these taxa were clustered with others from the fabids and malvids (Figure 6).

### 2.3. Orthology Analysis of NRT2 and NRT3

**Figure 7:**
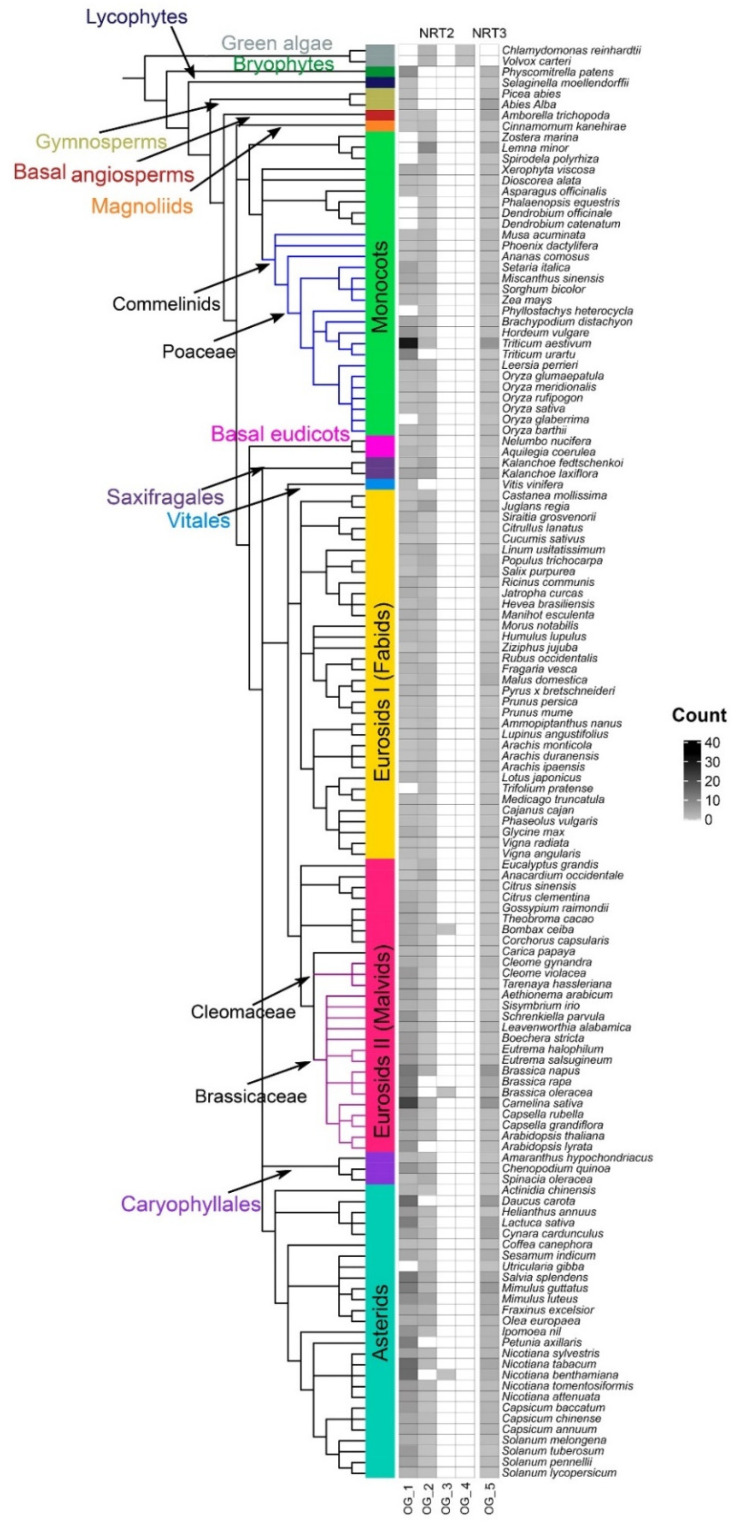
Heat map of orthologous groups (OG). Phylogenetic profile of the assigned OG within the NRT2 and NRT3 families in the 132 plant species studied. The color of the boxes indicates the number of sequences that make up an orthogroup in each species.

To discern the homologous protein sequences between the analyzed species, groups of orthologues or orthogroups (OG) were determined. The map shows five OG, of which the OG1–OG4 groups belong to the NRT2 transporters, and the OG5 group belongs to the NRT3 proteins (Figure 7, Table S5).

The orthogroups OG1 and OG2 accounted for the majority, containing 589 and 251 sequences, respectively. OG1 was present in bryophytes and gymnosperms and from the basal angiosperm, identified in most species up to the asterids. All Arabidopsis NRT2 genes were in OG1, except AtNRT2.5 and AtNRT2.7, as well as the OsNRT2.1 and OsNRT2.2 genes. In angiosperms, orthogroups OG1 and OG2 observed, with some exceptions. OG2 was observed in algae, and it was re-identified even in angiosperms. In this orthogroup, AtNRT2.5 and AtNRT2.7 as well as the rice genes OsNRT2.3 and OsNRT2.4 were identified. OG3 was only observed in three eudicot species, *Bombax ceiba*, Brassica oleracea, and *N. benthamiana*, while OG4 was exclusive to algae (Figure 7).

Regarding the family of transporters NRT3 (NAR2), a single grouping of 297 sequences was observed in the OG5 with all species since bryophytes, where the AtNRT3.1 and AtNRT3.2 genes were identified (Figure 7).

## 3. Discussion

### 3.1. Variation in the Number of NRT2 and NRT3 Sequences as a Potential Driver of Habitat Diversity with the Availability of Different N Sources in Vascular Plants

The activity of one component of HATS (NRT2 proteins) or two components (NRT2 and NRT3 proteins) supplants the activity of LATS (NRT1 proteins) when nitrate availability decreases [7], and this activity is responsible for the acquisition of nitrate under poor N environments.

Adverse conditions on the planet as well as fluctuations in nutrient availability have generated modifications in the genome of plants to achieve adaptation [50,51]. Although the members of the NRT2 and NRT3 gene families have been described for different plant species, to date, there is no study that evaluates the complete evolutionary history of this nitrate transport system in plants using a set of species belonging to a large number of taxa.

The number of NRT2 and NRT3 sequences found in this work (Figure 1) coincided with those previously reported in species such as *A. comosus* [33], *Z. mays* [34], *B. napus* [7], and *A. thaliana* [20], but this differed from other species [35,40,52].

A larger number of NRT2 sequences were detected in 18 of the 20 land plants previously studied by von Wittgenstein et al. (2014) [40]. These authors used first released versions of genomes, and therefore, the proteome annotations were immature; however, at present, the genomes have been better assembled, allowing the identification of more sequences in these species. In contrast, a lower number of NRT2 and NRT3 sequences than previously reported for green algae [52] and *H. vulgare* [35] was identified in this work. These discrepancies emerged because these proteins did not meet the criteria established in our work, in which possible functional NRT2 and NRT3 were selected for nitrate transport activity, screening candidate proteins with a lack of 40% or more of the established structure for HMM profiles.

Under this criterion, the only previously reported NRT3 sequence of *V. carteri* [52] was discarded here. Unlike the NRT3 protein of *C. reinhardtii*, which is known to be a component of HATS in this alga [53], that of *V. carteri* has not yet been functionally characterized, so it is unknown whether the lower conservation of this sequence influences the functionality of the protein. Likewise, NRT3 proteins are not present in a large number of algae species, suggesting that a single-component HATS could be efficient enough to guarantee cell growth and the functioning of these algae [52].

Our results confirmed that the NRT2 family was more abundant than NRT3 in the vast majority of species, as has been reported to date [33,34,35]. However, an important finding in this work was the detection of four species, the two Gymnosperms, *A. trichopoda*, and *D. officinalis*, with a higher number of NRT3 vs. NRT2 sequences (Figure 1).

From the lycophyta, a reduction by half of the number of NRT2 sequences was observed when compared with the ancestral green algae, and this reduction was maintained in the two species of gymnosperms and the basal angiosperm. However, the opposite occurred for the NRT3 genes in these last two lineages, where a significant expansion of the same was observed, which led to the presence of a greater number of NRT3 sequences relative to NRT2 in these species (Figure 1).

The expansion of NRT3 sequences in gymnosperms seems to be due to both recent WGD events and gene duplications, while in the basal angiosperm *A. trichopoda*, it could have occurred only due to gene duplications since this species is the only angiosperm that did not experience additional WGD [51,54,55].

The specific retention of these duplicates of NRT3 in species where the NRT2 family underwent a contraction seems to be exclusive to these more ancestral lineages of seed plants, which, with the exception of the monocotyledonous *D. officinalis*, was not conserved in evolutionary events later (Figure 1). This result suggests the value of carrying out functional and regulatory studies of these proteins in these biological models that have not been explored until now in relation to nitrate transporter proteins. Such studies will contribute to determining whether if the selective expansion of NRT3 vs. contraction of NRT2 preceded the evolution of exclusive innovations in two-component nitrate uptake, which contributed to the growth and development of these species under specific ecological conditions.

### 3.2. Variability Analysis into Monocots

The number of NRT2 sequences was particularly variable within the monocots (Figure 1). The greatest sequence expansion was observed in the Poaceae family, which is one of the largest within the angiosperms and includes bamboo, pasture, and cereal grasses [50], standing out to the hexaploid wheat *T. aestivum*. A large part of the species that make up this family has undergone a domestication process where artificial selection could have been favored from gene duplications that led to new characteristics and that have facilitated natural selection [56,57]. This is particularly relevant considering that several NRT2 transporters have been related to increases in the yields of these crops [24,57]. In a recent report, single nucleotide polymorphisms in NRT2 genes in *T. aestivum* were identified, and then, it was used to group wheat cultivars according to morphometric traits. The accumulated polymorphism was mainly due to random drift rather than selection, and the variation on those genes leads to significant differences in the NUE of wheat [41].

However, some members of the monocots experienced the greatest loss of NRT2 sequences that were observed among vascular plants, with only one sequence of this family being identified in these members (Figure 1). In all cases, they were species that present adaptations or specialized ecological strategies, with a preference for other sources of N instead of nitrate.

For example, *Z. marina* belongs to seagrasses, the only vascular plants that colonized that environment, because they experienced re-gain of genes from aquatic organisms and loss of other genes acquired by angiosperms in their genome, which led to structural and physiological adaptations required for their marine lifestyle [58]. The nitrate concentration in ocean water is very low [59], and it has been shown that under these conditions, a decrease in the cytosolic nitrate concentration occurs in *Z. marina* [37]. Rubio et al. (2019) reported induction of the only members NTR2 and NRT3 of this species, use of Na^+^ (in contrast to terrestrial plants that use H^+^ symporters for high-affinity NO_3_^−^ uptake), and dependent high-affinity nitrate transporter under conditions of N deficiency and high HCO_3_, suggesting that this induction is the main response during the decrease in cytosolic nitrate that occurs in *Z. marina* and might be an essential adaptation of seagrasses to colonize marine environments [37].

*P. equestris*, *D. officinalis,* and *D. catenatum* belong to the Orquidaceae family, where epiphytic or lithophytic lifestyles could be linked to the adaptive radiation of orchids [60]. These three species have evolved to adapt to these ecological conditions [61,62,63].

These species have aerial roots with large surface areas for rapid nutrient uptake. However, it has been shown that they are highly dependent on their mycorrhizal fungal partners for the acquisition of nutrients [64], with N likely being the main nutrient transferred to the plant by the fungus [65]. Although it is known that mycorrhizae can transfer different sources of N to terrestrial plants [66,67], it was recently shown that the cosmopolitan orchid mycorrhizal fungus *Tulasnella calospora* is incapable of using nitrate since it does not have transporter genes for the uptake of this ion in its genome. Strong expression of amino acid transporters occurs in both the fungus and the plant in a situation of symbiosis, suggesting that orchids largely acquire organic N [68,69].

On the other hand, the bamboo *P. heterocycla* is the only major lineage of grasses that is native to forests and whose rapid growth (“explosive growth”) of the shoots as well as the switch to flowering after a long period of vegetative growth are unique characteristics of this family [70]. This rapid growth is accompanied by high nutrient dynamics, and it has been determined that in the forest soils where these giant bamboos grow, the mineralization of N is dominated by ammonification, with ammonium being the main form of N in them [71]. Interestingly, these bamboos seem to present a species-specific strategy, with a high preference for ammonia, a rapid growth response to this source, and greater tolerance to the presence of high concentrations of this nutrient. These results suggest that in these forests, soil N is converted from a mixture of nitrate and ammonium to mostly ammonium during the invasion process of bamboo, where other plants cannot compete with “ammonium specialist” bamboo [72].

### 3.3. Variability Analysis into Eudicots

The highest concentrations of expansion events in both protein families appear to have occurred in the malvid and asterid groups (Figure 1). The increase in the number of sequences in several Brassicaceae can potentially be the product of WGD events in this lineage [73]. Similarly, several rounds of WGD and WGT have been identified in the asterids, which has been associated with rapid radiation that has coincided with dramatic climate changes, linking polyploidies to survival in a period of ecological and environmental crisis [74]. This last lineage is highly diverse in ecology and morphology.

Within the asterids group, *U. gibba* stands out, and despite having suffered a recent WGD event, an expansion of NRT2 sequences was not observed when compared to the number of sequences in ancestral vascular plants. This result indicates that this species was not able to retain the NRT2 genes after the duplication event, suggesting the contribution of environmental adaptations. This species is aquatic and carnivorous, which is a syndrome that has evolved as a specialized solution to acquire nutrients in N- and P-limited environments. In addition, it presents a highly specialized body plant, including the absence of a root in it [75].

The expansion of gene families and functional diversification may have contributed to the adaptations required for life in terrestrial habitats [76], where the nitrate concentration is highly variable, both vertically and horizontally. The expression of the different copies of genes NRT2 and NRT3, specific for tissues and N conditions, in crops, such as rice [31], corn [34], pineapple [33], barley [35], rapeseed [7], and arabidopsis [23], among others, suggests that the presence of a greater number of these sequences in their genome could be a source of variation for the evolution of innovations of these plants to occupy niches with contrasting N sources and concentration conditions.

The diversity in the use of N among plant species represents a history of more than 350 million years of evolution, where the species have evolved in continuous strategies of use of N with adjustments of the conservative and competitive mode, depending on availability [77]. The variation in the N content of the soil is an important source of selection for genes that can generate functional traits associated with certain plant growth strategies [78].

Although it is known that many environmental and physiological factors can influence the preferences of a source of N in plants [79,80], the variability in the number of NRT2 sequences within vascular plants detected in this work and the previous knowledge of the preferences for N sources in some species clearly suggests that the plants evolved genetic adaptations (for example, loss of NRT2 sequences in gymnosperms, epiphytes, and carnivores) for the acquisition of N-specific sources and can be found in niches enriched with that source of N for which they are best adapted.

### 3.4. Phylogenomic and Microsynteny Analysis of NRT2 and NRT3 Gene Families in Angiosperms

The combined analysis of phylogeny and synteny constitutes a valuable tool for the study of gene families in a large number of species. Our results demonstrated that approximately 40% and 30% of the NRT2 and NRT3 genes, respectively, did not form syntenic networks, implying specificity in genomic contexts that could indicate different regulatory and functional mechanisms of these proteins in these species.

No syntenic relationships were found for either protein family in the green algae, bryophytes, or the most ancient vascular plants, the lycophytes and gymnosperms (Figure 3, Figure 5 and Figure 6). Although it is known that all the NRT2 and NRT3 characterized to date are functional for the transport of high-affinity nitrate [15], important changes in mechanisms of regulation of the same between these ancestral organisms and seed plants have been reported [52,80,81]. Our data suggest that these regulatory differences could be a consequence of the location of these genes in genomic contexts that differ from angiosperms.

In *C. reinhardtii*, whose preferred source of N is ammonium, the NRT2 genes are grouped into two different clusters, and this grouping seems to facilitate the coordination of their regulation, where marked differences have been detected at the transcriptional and posttranslational level with those of higher plants [52]. For example, ammonium removal is necessary for nitrate-dependent induction of nitrate reductase transcription to occur, unlike higher plants, where this induction is independent of ammonia [52,82].

For the moss *P. patens*, although it is known that its NRT2 and NRT3 sequences are more closely related to those of plants than to those of green algae, it has been reported that the transcription of these genes is completely inhibited by ammonium, with glutamine being the signal metabolite responsible for this inhibition [81]. Instead, the inhibition by ammonium of AtNRT2.1 from *A. thaliana* is partial and occurs both by ammonium and glutamine [83]. In our work, all the NRT2 and NRT3 sequences of *P. patens* were grouped in clades basal to vascular plants, where multiple duplication events were observed, while for the lycophytes, a single duplication event seems to have occurred for NRT2 (Figure 5 and Figure 6).

Conifers are species that grow better in the presence of ammonium and organic N than nitrate [80]. Nitrate transporters have not been studied in these species, but it has been shown that the induction of the nitrate acquisition apparatus by the appearance of nitrate in the external environment can take several days in them, a surprising difference with the vast majority of the angiosperms studied, in which this induction usually happens very quickly, even within hours [7,34,35,80]. Likewise, all the NRT3 sequences of these species were grouped in a basal clade to the seed plants, indicating multiple duplication events, both in *P. abies* and in *A. alba* (Figure 6).

It would be interesting to explore NRT2-like sequences from other organisms that are capable of assimilating nitrate, such as bacteria, yeasts, and fungi [15], to search for possible orthologous genes between these and green algae and ancestral terrestrial plants, which were negative in terms of synteny in our study. The grouping of genes related to the absorption and assimilation of nitrate seems to be common in these organisms [84,85], where, for example, in fungi, it is associated with fungal virulence and with pathways that confer some advantage over competitors during evolution [86,87]. In plants, although the reason for this grouping is not yet understood, it seems to occur more frequently than expected [88,89].

In Angiosperms, it is noteworthy that NRT2 and NRT3 genes of some species were not located in synteny communities, indicating that they modified their genomic context during evolutionary processes. Such is the case for the freshwater duckweed *L. minor*, in which a great expansion of sequences was observed for both protein families (Figure 5 and Figure 6).

The data indicated multiple duplications of *L. minor* NRT2 and NRT3 genes, which coincides with previous reports that more than 61% of its genome contains repeated sequences and a greater number of transport-related genes than the major freshwater duckweed *S. polyrhiza*. It is interesting that this aquatic macrophyte, adapted to a wide variety of climatic regions, also has a greater number of genes that encode enzymes related to N assimilation and that regulate important biochemical pathways so that this species can remove excess nutrients from wastewater [90]. Based on these results, the NRT2 and NRT3 proteins of these species could constitute potential candidates for functional studies as well as for understanding the evolution of fundamental innovations within terrestrial plants, including tolerance to deficits or excess nutrients.

The phylogenetic reconstruction of the NRT2 family suggests its separation into two large clades that coevolved from seed plants (Figure 5). Von Wittegensteins et al. also suggested this divergence; however, they could not resolve the time in which it occurred since members of the gymnosperm and basal angiosperms were not included in their work [40]. Contrary to what these authors reported, clade I (in their work named clade II) was represented by all the species of plants with seeds included in our study except the member of the vitales, so the loss of genes in this group appears to be uncommon during the evolution of seed plant species (Figure 5). However, for members of clade II, two contrasting processes appear to have occurred during evolution. On the one hand, no sequences were found in a greater number of species (nine), indicating that the loss of genes during the evolution of seed plants may be more common in this clade. However, a very significant increase in the number of sequences in lineages, such as the monocots and various eudicots (fabids, malvids, and asterids), was also observed in this clade (Figure 5).

Interestingly, the species that did not have NRT2 members in clade II were those plants that evolved to specialized ecological strategies, such as plant with aquatic lifestyle, epiphytes, and carnivores. Additionally, within these species were the forage legume *T. pratense*, one of the most important in temperate agriculture, which is capable of fixing atmospheric N [91], and the African rice *O. glaberrima*, which has an origin other than Asian rice (*O. sativa*), which may not show the significant variations that *O. sativa* has in its preference for N source [18,89,92]. These results suggest that in the specialized adaptive process that these species underwent, they only retained NRT2 sequences from clade I, while they lost those belonging to clade II. In turn, the expansion in the number of genes in this clade versus clade I occurred for species belonging to the largest angiosperm families and highly diversified in ecology and morphology, with highly contrasting terrestrial habitats [50,55,74].

**Figure 8:**
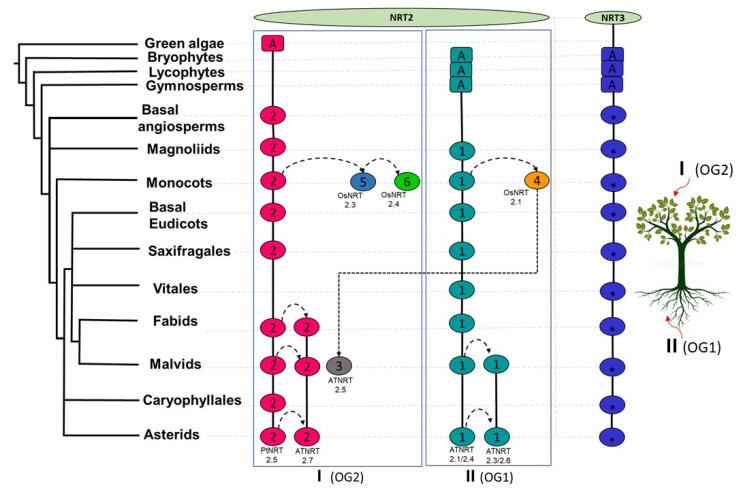
Proposed model of the evolutionary history of nitrate transporters NRT2 and NRT3. Syntenic communities are represented in ovals, using colors similar to those of Figure 5 and Figure 6 for NRT2 and NRT3, respectively. The rectangles represent ancestral genomic contexts (A). I, Clade I; II, Clade II; OG1, orthogroup 1; OG2, orthogroup 2; Pt, *P. trichocarpa*; At, *A. thaliana*; Os, *O. sativa*.

Taking into account the NRT2 sequences previously characterized in species, such as *O. sativa* [19,31], *H. vulgare* [35], *Z. may* [34], *A. thaliana* [93], *B. napus* [7], *A. comosus* [33], and *P. trichocarpa* [32], clade I grouped those proteins that participate in long-distance nitrate transport and were mainly expressed in organs of the aerial part. On the other hand, clade II grouped those that participate in the uptake of nitrate by the root. Both clades also presented different genomic architectures, observing more dynamic patterns of synteny between the subclades that constitute clade I (Figure 5). Com2NRT2 was the most ancestral and was identified from the basal angiosperm (Figure 8). From it, connections between syntenic pairs belonging to the different subclades of clade I were identified; this result suggests that the functions of these sequences are facilitated by a shared genomic context. The same occurred for the syntenic pairs that belonged to Com1NRT2, within clade II, in which interactions between intra- and inter-subclade syntenic pairs were identified, as found in the magnoliids (Figure 8).

Taking these analyses together, it is suggested that the conservation of these two major genomic contexts, Com2NRT2 and Com1NRT2 in clades I and II, respectively, could indicate that long-distance nitrate transport processes as well as the remobilization of this nutrient between aerial organs could be correlated with the most ancestral genomic context, that of Com2NRT2, while nitrate uptake by the root could reassemble with the Com1NRT2 genomic context.

However, for the first time, lineage-specific syntenic relationships within the NRT2 family were defined in this work. Within clades I and II, the vast majority of the monocot and eudicot proteins were grouped in different syntenic blocks, which implies different regulation of nitrate uptake processes in these two major angiosperm lineages (Figure 5). This is remarkable, particularly in the lineage of grasses, where the majority of the genes in synteny were located in specific syntenic communities for this lineage, such as Com5_NRT2, Com6_NRT2, and Com4_NRT2, with the first of those genes grouped in clade I and the last in clade II (Figure 5). These results are supported by what was previously reported, where it was discovered that none of the NRT2 genes of grasses had introns, unlike those of eudicots, indicating an ancestral divergence of the members of this gene family [94].

In Com5_NRT2, orthologues of AtNRT2.5 were found in the monocots *O. sativa* (OsNRT2.3) [25,95], *H. vulgare* (HvNRT2.1) [35], and *Z. may* (ZmNRT2.5) [34], differing in genomic context with from their *A. thaliana* orthologue, which was identified in Com3NRT2 (Figure 5).

This result may contribute to understanding the differences in the expression patterns of NRT2.5 between monocots and eudicots that have been reported. For monocots, this gene, in addition to being expressed in roots and leaves, as in eudicots, is also expressed in embryos and shells in wheat [57,96] and in silk, cobs, and tassel husk leaves in corn [34,97]. Eudicot expression has only been found at extremely low levels in dried *Arabidopsis* seeds [18] but not in poplar [32], teatree [98], or cassava [25]. Thus, in addition to the important role of NRT2.5 in Arabidopsis during N starvation and absorption of nitrate at very low concentrations, in monocots, it plays a fundamental role in the accumulation of nitrate in the seed and the filling of the grain [25,34,57,99].

Similarly novel, it turned out that the AtNRT2.5 and AtNRT2.7 sequences of *A. thaliana* did not share genomic contexts. Unlike AtNRT2.5, which appears to be involved in various functions throughout the plant, as previously described, AtNRT2.7 has been identified in seeds, including the embryo and seed coat, and has a specific function in nitrate storage in the seed, being important for its germination [18].

Within the subgroups G5 to G7 belonging to clade II, no previously characterized sequences were identified; however, numerous syntenic connections within and between these subclades and with subclades G8 and G9 were observed. A potential shared function between these sequences remains to be demonstrated.

In the G8 subclade, the Arabidopsis proteins that are responsible for nitrate uptake by the root, AtNRT2.1, AtNRT2.2, and AtNRT2.4, were grouped (Figure 5). AtNRT2.1 and AtNRT2.2 are neighboring genes in opposing orientations [94], and AtNRT2.1 is the protein that carries out 80% of high-affinity nitrate uptake in the root, while AtNRT2.2 can replace its function under conditions of loss of AtNRT2.1 activity [23,24]. In contrast, AtNRT2.4 has a particularly high affinity for nitrate, suggesting that it participates in the acquisition of nitrate when the concentrations in the medium are very low [100]. In this subclade, the seven BnNRT2.1 sequences and the two of BnBRT2.2 from *B. napus* were also grouped. It has been reported that extensive BnNRT2.1 appears to be the product of allopolyploidy or duplication events in the evolutionary history of these plants, mainly generated by WGD events. These duplications appear to have led to divergence of specific roles of these proteins in response to variable nutritional conditions [7].

Interestingly, the G9 subclade grouped sequences from the most recent lineages, mainly from malvids and asterids, and here, the two remaining sequences from *A. thaliana*, AtNRT2.3 and AtNRT2.6, were identified (Figure 5). AtNRT2.3 and AtNRT2.4 have been reported to be tandem repeat genes, while AtNRT2.6 is located on a completely separate chromosome [94]. These proteins have been less studied although their function as nitrate transporters has been confirmed for AtNRT2.3 [23] but not for AtNRT2.6. The latter, together with AtNRT2.5, plays an essential role in the regulation of growth in response to rhizospheric bacteria, an effect that is independent of nitrate [27]. Likewise, in this subgroup, two BnNRT2.3 sequences were identified, whose functional activity has not been characterized. These results suggest that although all these genes preserved synteny, the expansion of this family may have been a source of variation for the evolution of the function of these genes and biological innovations in these species.

NUE is a complicated agronomic trait because it involves the multiple interconnected steps of nitrate assimilation, transport, and signaling. In fact, some transgenic developments have shown that it is promising to manipulate the expression of genes involved with N transport to improve cultivation and agricultural production [19,101]. From our study, analytical tools, such as Genome-Wide Association Scan (GWAS) and Genome-Wide Selection Scann (GWSS) [102], could be used to reconstruct the divergent genomic architecture of adaptative traits under variable nutriment conditions. The importance of these studies was demonstrated in a recent study of GWAS developed in wild and domesticated accessions of barley, allowing to identify markers trait associations conferring efficient nitrogen use. HvNRT2.7 was identified as a promising candidate to increase the dry weight of the root and shoot and became a potential target for genetic improvement in this species [103]. In addition, for the pleiotropic effects of the NRT2 and NRT3 gene families, recent findings indicate that the genetic basis of efficient transport of nitrate involves these transporters among a pyramid of several genes that must be ranked to achieve the most effective approach that allows a satisfactory NUE level in the field [104] In rice crops, it has been shown that the overexpression of the OsNRT2.3b transporter increases the NUE, but this response improves if this transporter is introduced together with the promoter OsNAR2.1p. Since it increases the NUE level and the yield of the grain, it additionally influences the homeostasis of the pH, which improves the absorption of ammonium, P, and Fe as well as the metabolism of other nutrients [19,101].

For the case of the NRT3 family, our results suggest the existence of a strict positional restriction to preserve the function of NRT3 since it displayed conservation of synteny throughout all the angiosperm species evaluated (Figure 6). For example, in *C. reinthardii*, it has been shown that CrNRT2.1/CcNRT3 constitutes the bi-specific nitrate/nitrite high-affinity transport, while CrNRT2.2/CcNRT3 only transports nitrate [53]. In *Z. may*, ZmNRT2.1 is part of a tetramer with ZmNRT3.1 and is the functional unit responsible for HATS in the root of this species [105]. Likewise, OsNRT3.1 from *O. sativa* was required for the nitrate transport of OsNRT2.1, OsNRT2.2, and OsNRT2.3a, while all the NRT2s from *A. thaliana* except AtNRT2.7 interact with AtNRT3.1 [18].

The orthology analysis of the NRT2 family showed that the most ancestral OG corresponded to OG2, which presumably originated from green algae and where all the genes that belonged to clade I were identified, with some exceptions. In contrast, OG1 originated from terrestrial plants, encompassing all sequences belonging to clade II (Figure 7). This result suggests for the first time that the ancestors of the NRT2 proteins that participate in long-distance transport and nitrate remobilization in aerial organs and those that function in the root were different, being that of the most ancestral aerial part. Instead, these analyses showed that the NRT3 family of all land plants had a common ancestor, and this ancestor apparently arose from mosses (Figure 7).

The evolutionary history traced in this work is based on the microsynteny and orthology analysis using NRT2 and NRT3 genes from 132 genomes. This allows to establish a more precisely the internship between transporters from various lineages. However, it is necessary to apply other strategies, such as genomic prediction and mapping of changes through massive analysis of SNPs [41], in the context of the environment of each genotype and understand the effect of the relationship genotype environment exerts on the selection of genetic variants in the intake and transport of nitrate.

The knowledge of the genomic context of NRT2 in genotypes with contrasting habitats and domestication processes could make it possible to identify key genomic signatures of adaptation and domestication. Therefore, it can offer the potential to improve nutrient use efficiency and tolerance to adverse environmental conditions through naturally existing genetic variants [106]. The data generated in this work can be incorporated into genomic prediction and machine learning studies. Therefore, it can help understand the bases of the genetic adaptations of species in response to climate change and environmental stress [102,106,107,108] since the absorption of N can act as a factor that enhances or limits these adaptive responses [78,109].

## 4. Materials and Methods

### 4.1. Database Construction

One hundred thirty-two plant species (*Sensu lato*) were analyzed, including green algae, bryophytes, lycophytes, gymnosperms, and angiosperms (Table S1). Protein sequences from fully sequenced genomes as well as GFF/GFF3 attachments were downloaded from the Phytozome net (v11, Berkeley, CA, USA), SolGenomics net (National Science Foundation, USA); PepperGenome (Next-Generation BioGreen, v1.55, Daejeon, Korea); Ensembl (ensembl.org (accessed on 14 October 2021), EMBL-EMI, Cambridgeshire, UK); NCBI (https://www.ncbi.nlm.nih.gov/ (accessed on 14 October 2021), USA), and Norway Spruce databases (wood-database.com (accessed on 14 October 2021)) (Table S1).

Hidden Markov models (HMM) profiles were built by HMMER 3.1b package (https://hmmer.org/; last accessed 7 May 2020) and used for the search of NRT2 and NRT3 (NAR2) proteins. Additionally, HMM from the Pfam database [110] was used. A custom NRT2.2 HMM profile was built to search for proteins containing the NRT2 domains while that of the NAR2 domain (PF16974.5) was for NRT3 proteins. Proteins that met the profile-specific gathering threshold (option (--cut_ga) of hmmsearch) and a domain coverage equal to or greater than 60% were considered for the analysis of synteny and orthology. The recovery of the sequences was carried out with the seqret program of the EMBOSS 6.6.0.0 package [111].

### 4.2. Phylogenetic Inference

Multiple sequence alignments (MSA) were carried out with MAFFT v7.407, using the FFT-NS-2 option [112,113]. Alignments containing 20% or more gaps were removed with TrimmAI v1.2rev59 [114]. Additionally, a manual edition was carried out with UGENE v1.31.0 [115] to keep only the region of the domain of interest for each protein family. The phylogenetic trees were built with IQ-TREE v2.0.6 [116,117]. For each MSA, the best amino acid substitution model (WAG, LG or JTT) was chosen according to the Bayesian information criterion (BIC) with ModelFinder [118] and implemented in IQ-TREE. The “UltraFast Bootstraps” (UFBoot) tests [119] and the SH-aLRT test (1000 replicas each) were implemented, within the IQ-TREE program to obtain branch support values. The phylogenetic trees with all the sequences were constructed with FastTree v2.1.11 [120,121] and visualized with iTOL v5.6 [122] and ggtree v2.3.2 [123].

### 4.3. Synteny Analysis

A microsynteny network approach was implemented in which protein-encoding genes were represented as nodes and the edges or connecting lines between nodes as pairwise synteny relationships, following the process described at https://github.com/zhaotao1987/SynNet-Pipeline (last accessed 7 May 2020) [43,124] considering five genes per side flanking the NRT genes. This analysis was carried out in two stages. First, a global synteny network was generated from all the protein-coding genes contained in the dataset of the 132 proteomes. Reciprocal similarity searches were performed, with RAPSearch v2.24, options: (-b 0 -v 20 -t a -p f -a t) [125]. The detection of syntenic pairwise relationships was carried out with MCScanX [126]. In the second step, protein sequence identifiers were used to retrieve all pairwise synteny relationships within the NRT2 and NRT3 families of the entire microsynteny network. In each protein family, communities of collinear sequences were established with the Infomap clustering algorithm [127,128] implemented in the igraph v1.2.4.1 package [129] in R v3.6.3. Communities were considered the groupings of genes present in three or more species (sp_n ≥ 3). Networks and communities were visualized with Cytoscape v3.8.0 [130] and Gephi v0.9.2 [131], respectively. The phylogenetic profile of the detected network communities was visualized using the ComplexHeatmap v2.5.3 package [132] in R v3.6.3 [133].

### 4.4. Orthology Analysis

For each species, the complete sets of NRT2 and NRT3 protein sequences were concatenated into a single file. Subsequently, the proteins were assigned to orthogroups using the Broccoli v1.2 program [134], which combines phylogeny and network analysis. The broccoli options were: (-sp_overlap 0.3-e_value 1^−20^); Diamond v0.9.31.132 [135] and FastTree v2.1.11 [120,121] were implemented in Broccoli v1.2. The phylogenetic profile of the orthogroups was visualized through a heat map with the ComplexHeatmap v6.2.3 package [132].

## 5. Conclusions

In this study, we generate a broad-scale phylogenomic analysis for NRT2 and NRT3 gene families, which contributes to updating the knowledge and understanding the evolutionary history of these HATS in land plants. Based on the phylogenetic analysis of NRT2, we propose the existence of two main clades, I and II, which coevolved from gymnosperms, the first arising from green algae and the second from bryophytes. The great dynamics that were identified in the genomic contexts between these two clades may help to understand the differences in the regulation of the expression of these genes in specific organs, for example, in the aerial part (clade I) or in the root (clade II) as well as between monocots and eudicots. It is suggested for the first time that the specialized adaptation and lifestyle of some species (aquatic lifestyle, epiphytism, and carnivory) is a factor that strongly contributed to the loss of NRT2 genes during the evolution and have led to changes in preferences for the acquisition of other forms of N instead of nitrate. These plants retained the genes that clustered in clade I but lost those in clade II. A higher expansion of NRT2 genes was identified in clade II and correlated with lineages that present high radiation and are capable of growing under nutrient-contrasting soils. NRT2’s partner, NRT3, conserved the genomic context throughout the angiosperms, which points to conservation of the regulation of these proteins, having mosses as its ancestor. This work lays the foundations for the first time for the study of potential candidates that can contribute to increasing the development and efficient use of N in species of interest and opens the door to the knowledge of innovative mechanisms of specific-lineages high-affinity nitrate absorption. Our results, in combination with phylogeography, can be used in genomic prediction models to infer transgenerational adaptive traits and identify genetic markers that could be used in breeding programs to increase crop productivity and adaptability face to environmental changes.

## Data Availability

All relevant data are within the manuscript and its Supporting Information file.

## Supplementary Materials

The following are available online at https://www.mdpi.com/article/10.3390/ijms222313036/s1.
