# Supplementary figures and images for: Phylogenomic and Microsynteny Analysis Provides Evidence of Genome Arrangements of High-Affinity Nitrate Transporter Gene Families of Plants
Source: Int J Mol Sci. 2021 Dec 3;22(23):13036. doi: 10.3390/ijms222313036 (PMC8658032; doi:10.3390/ijms222313036)

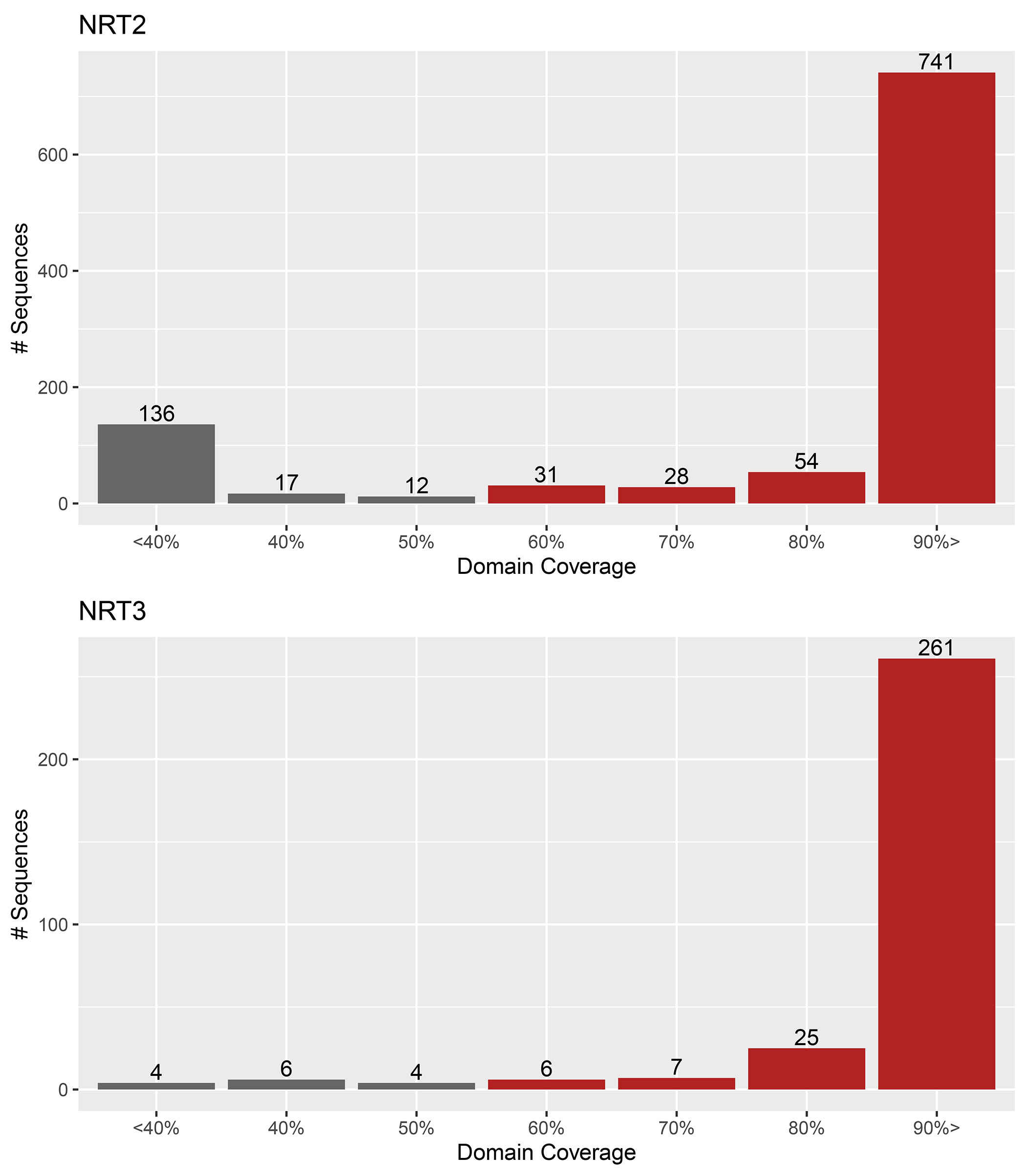

Supplement: Supplementary file 1 [file ijms-22-13036-s001.zip › FigureS1.jpg]

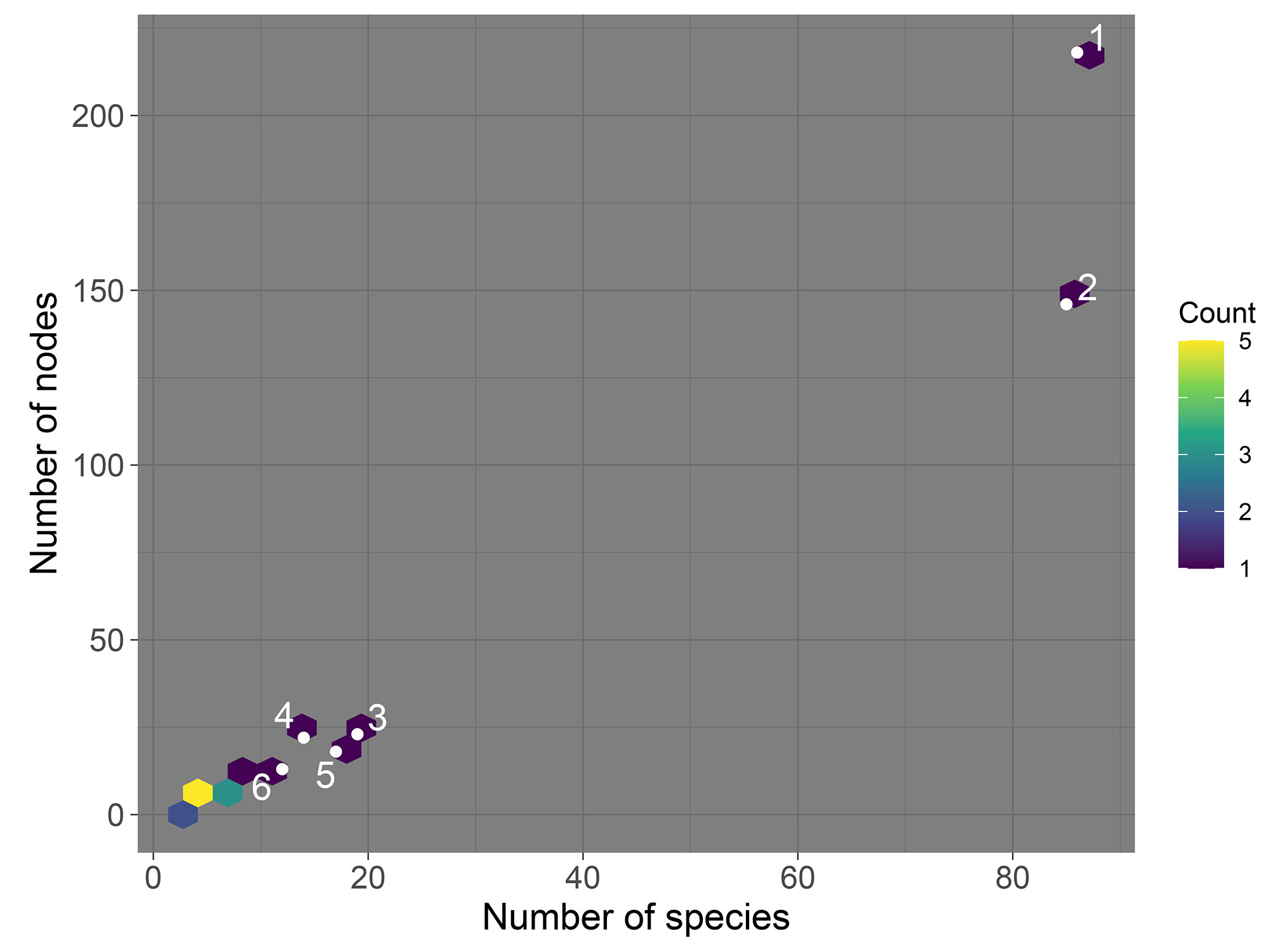

Supplement: Supplementary file 1 [file ijms-22-13036-s001.zip › FigureS2 .jpg]
